# A national population‐based cohort study to investigate inequalities in maternal mortality in the United Kingdom, 2009‐17

**DOI:** 10.1111/ppe.12640

**Published:** 2020-02-03

**Authors:** Marian Knight, Kathryn Bunch, Sara Kenyon, Derek Tuffnell, Jennifer J. Kurinczuk

**Affiliations:** ^1^ National Perinatal Epidemiology Unit University of Oxford Oxford UK; ^2^ Institute of Health Sciences University of Birmingham Birmingham UK; ^3^ Bradford Hospitals NHS Foundation Trust Bradford UK

**Keywords:** cohort analysis, ethnic groups, maternal age, maternal mortality, socio-economic factors

## Abstract

**Background:**

Disparities have been documented in maternal mortality rates between women from different ethnic, age and socio‐economic groups in the UK. It is unclear whether there are differential changes in these rates amongst women from different groups over time. The objectives of this analysis were to describe UK maternal mortality rates in different age, ethnic and socio‐economic groups between 2009 and 2017, and to identify whether there were changes in the observed inequalities, or different trends amongst population subgroups.

**Methods:**

Maternal mortality rates with 95% confidence intervals (CI) in specific age, deprivation and ethnic groups were calculated using numbers of maternal deaths as numerator and total maternities as denominator. Relative risks (RR) with 95% CI were calculated and compared using ratios of relative risk. Change over time was investigated using non‐parametric tests for trend across ordered groups.

**Results:**

Women from black and Asian groups had a higher mortality rate than white women in most time periods, as did women aged 35 and over and women from the most deprived quintile areas of residence. There was evidence of an increasing trend in maternal mortality amongst black women and a decrease in mortality amongst women from the least deprived areas, but no trends over time in any of the other ethnic, age or IMD groups were seen. There was a widening of the disparity between black and white women (RR 2.59 in 2009‐11 compared with 5.27 in 2015‐17, ratio of the relative risks 2.03, 95% CI 1.11, 3.72).

**Conclusions:**

The clear differences in the patterns of maternal mortality amongst different ethnic, age and socio‐economic groups emphasise the importance of research and policies focussed specifically on women from black and minority ethnic groups, together with other disadvantaged groups, to begin to reduce maternal mortality in the UK.

**Synopsis**

**Study question**

Are there differences in maternal mortality rates amongst women from different ethnic, age and socio‐economic groups in the UK and are these differences changing over time?

**What is already known**

The maternal mortality rate overall has been static in the UK for more than 10 years. There are known differences in mortality rates between women from different ethnic, age and socio‐economic groups in the UK, but changes in mortality rates over time amongst different population subgroups have not been examined.

**What this study adds**

This study shows that maternal mortality amongst black women in the UK is increasing, with evidence of a widening inequality between black women and white women. Maternal mortality amongst women living in less deprived areas is decreasing.

## 1 BACKGROUND

Maternal mortality surveillance has been conducted in the UK since the early 1950s, but the methods and focus of reports have evolved over time. The epidemiology of maternal mortality has almost completely changed from two‐thirds of deaths being due to direct obstetric causes in 1952‐54,^1^ to almost two‐thirds being due to medical and mental health‐related causes in 2015‐17.^2^ Enhanced case ascertainment commenced at the turn of this century,^3^ allowing in particular more robust identification of indirect, medical or mental health related, maternal deaths. Enhanced ascertainment, which is still only carried out in a few countries, thus allows reliable calculation of true maternal mortality rates both for the whole population and amongst different population subgroups. Disparities have subsequently been documented in maternal mortality rates between women from different ethnic, age and socio‐economic groups. Women who were older, or who had more deprived backgrounds, or who were from black or other minority ethnic groups were found to have higher rates of maternal mortality once cases identified through these enhanced processes were included.^4^ The last of the three‐yearly reports, reporting maternal deaths for 2006‐08, prior to change to annual reporting,^4^ suggested that maternal mortality rates amongst black women were decreasing at a more rapid rate than those amongst white women, against a background of a statistically significant overall decrease in maternal mortality. No assessment was made in this report for deaths in 2006‐08, however, of whether there were differential changes in the maternal mortality rates amongst women from different age or socio‐economic groups over time.

Maternal mortality rates in the UK have largely been static over the period 2009‐17,^2^ although globally maternal mortality ratios have decreased following the intense focus of the Millennium Development Goals^5^ and subsequent Sustainable Development Goals.^6^ Concerns have been raised recently in both the USA^7^ and Canada^8^ that maternal mortality rates are potentially increasing, although it has been suggested that the observed trend in the USA simply represents better case identification.^9^ Nevertheless, marked disparities have also been documented in mortality rates amongst women from different racial backgrounds in the USA.^10^ Whilst this analysis suggests these disparities did not change in the USA between 2007 and 2016, it is not clear whether disparities are changing with time in other countries.

From the 2009‐11 triennium onwards, maternal mortality data have been published on an annual basis in the UK, presented as rolling 3‐year average rates. The report published in 2018, including data for 2014‐16, highlighted the wide disparity in mortality rates between black, Asian and white women.^11^ The aim of this analysis was to describe maternal mortality rates in different age, ethnic and socio‐economic groups between 2009 and 2017 in the UK, and to identify whether there were any changes in the observed inequalities, or different trends amongst different population subgroups, in order to inform targeted actions to prevent maternal deaths in the future.

## 2 METHODS

This analysis is based on data extracted from published maternal mortality reports.^2^, ^11^, ^12^, ^13^, ^14^, ^15^, ^16^ Maternal deaths in the UK were identified through a variety of sources, as described in detail elsewhere.^14^, ^17^ In brief, deaths were reported directly to MBRRACE‐UK office by staff in the hospitals where women died, or through pathologists, coroners, procurators fiscal, other hospital staff or members of the public. They were also identified directly through media reports. Additionally, information on deaths of women who were reported to be or have been pregnant and/or have a pregnancy‐related cause of death was obtained from vital registration data from the Office for National Statistics (England and Wales), National Records Scotland or the Northern Ireland Statistics and Research Agency. Data on deaths amongst women of reproductive age were additionally linked to birth records from the previous year to identify deaths of any women who had given birth in the previous year but for whom no recent pregnancy or pregnancy‐specific diagnosis was recorded on their death certificate. All maternal deaths identified were classified according to the International Classification of Diseases—Maternal Mortality (ICD‐MM).^18^ Details about women's age, self‐reported ethnic group and postcode of residence were extracted from their medical records. Ethnic group was classified according to the UK census classification into white, black, Asian, Chinese/other and mixed.^19^ The area deprivation score (Index of Multiple Deprivation, IMD) for the super output area of women's postcode of residence was determined using a national look‐up table.^20^ The IMD is derived from 37 different indicators divided into seven different domains: Income Deprivation; Employment Deprivation; Health Deprivation and Disability; Education, Skills and Training Deprivation; Crime; Barriers to Housing and Services; and Living Environment Deprivation. These scores are used to rank the residential areas in England into five equal groups, or quintiles, from the least deprived 20% (quintile I) to the most deprived 20% (quintile V).

Denominator data were obtained from published data from the Office for National Statistics, National Records Scotland, Northern Ireland Statistics and Research Authority, NHS Digital, or from national birth data. Denominator maternities (women giving birth to a liveborn baby or a stillborn baby greater than or equal to 24 weeks' gestation) data classified by ethnic group and by area deprivation score were only available for England, and therefore analyses investigating ethnic group and socio‐economic deprivation were conducted only for England. Additionally, denominator data in the five census classification groups were only available from 2012; therefore, ethnicity data for mixed and Chinese/other groups are only presented from 2012 to 2014 onwards. Maternities for which ethnicity was not stated were included in the “white European” group because the re‐distributed proportions matched with the estimated ethnic profiles in the UK population census.^21^

### 2.1 Statistical analysis

Maternal mortality rates with 95% confidence intervals were calculated using numbers of maternal deaths as the numerator and total maternities in each specific group as the denominator. Relative risks with 95% confidence intervals were calculated to compare maternal death rates between groups of women. A non‐parametric test for trend across ordered groups was used to investigate the change in three‐yearly rolling maternal mortality rates over time.^22^ Estimated ratios of relative risk (RRR) were calculated to compare maternal death rates in the different age, socio‐economic and ethnic groups.^23^ Data were analysed in stata version 15 (Statacorp).

### 2.2 Ethics approval

No approval was required for this secondary analysis of published data.

## 3 RESULTS

**Figure 1:**
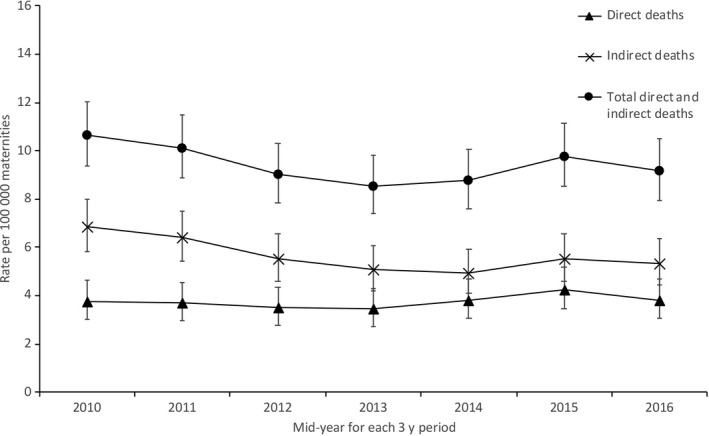
Maternal mortality in the UK 2009‐17

During the study period, there were 662 maternal deaths amongst 7 001 210 maternities; an overall maternal mortality rate of 9.46 per 100 000 maternities (95% CI 8.75, 10.20). There was no difference in overall maternal mortality rate in the UK between 2009‐11 and 2015‐17 (RR 0.86, 95% CI 0.72, 1.04; Figure 1), and no evidence of a trend over time.

**Figure 2:**
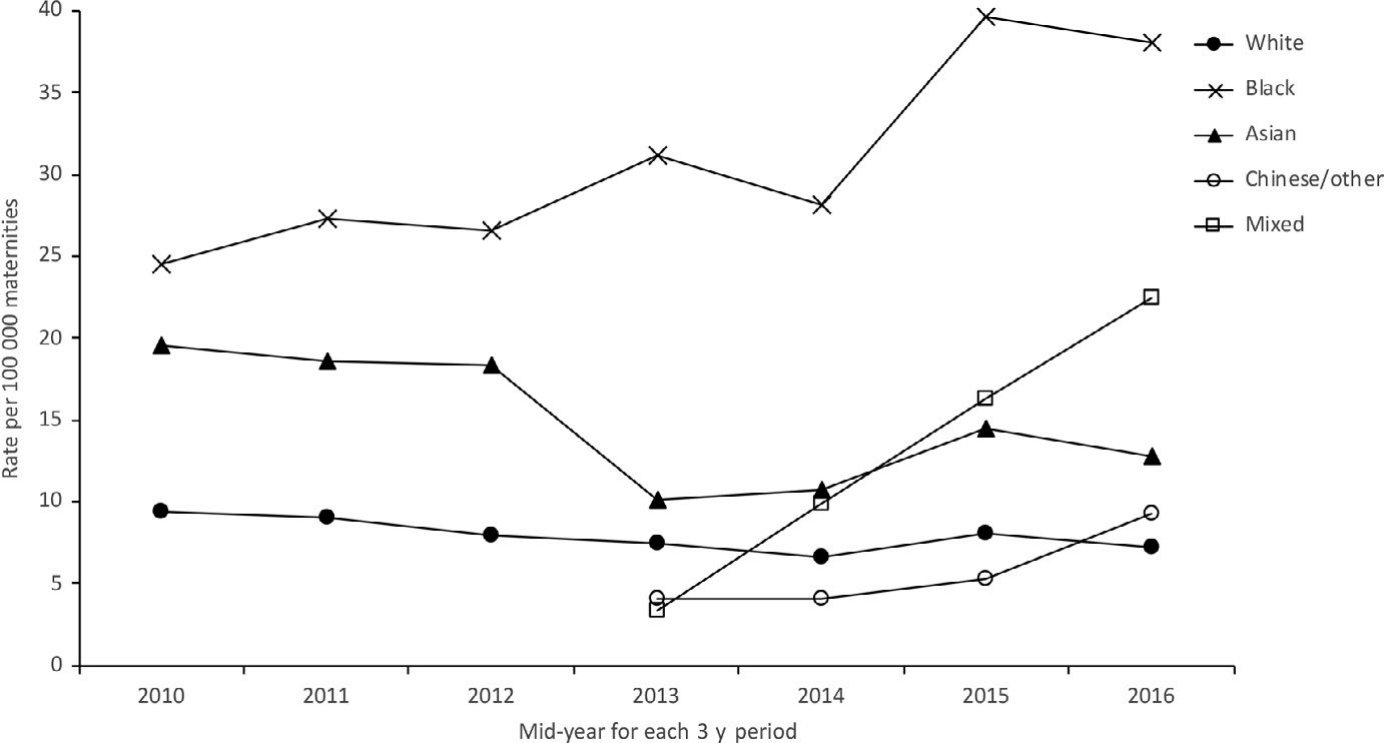
Maternal mortality amongst different ethnic groups in England 2009‐17

**Figure 3:**
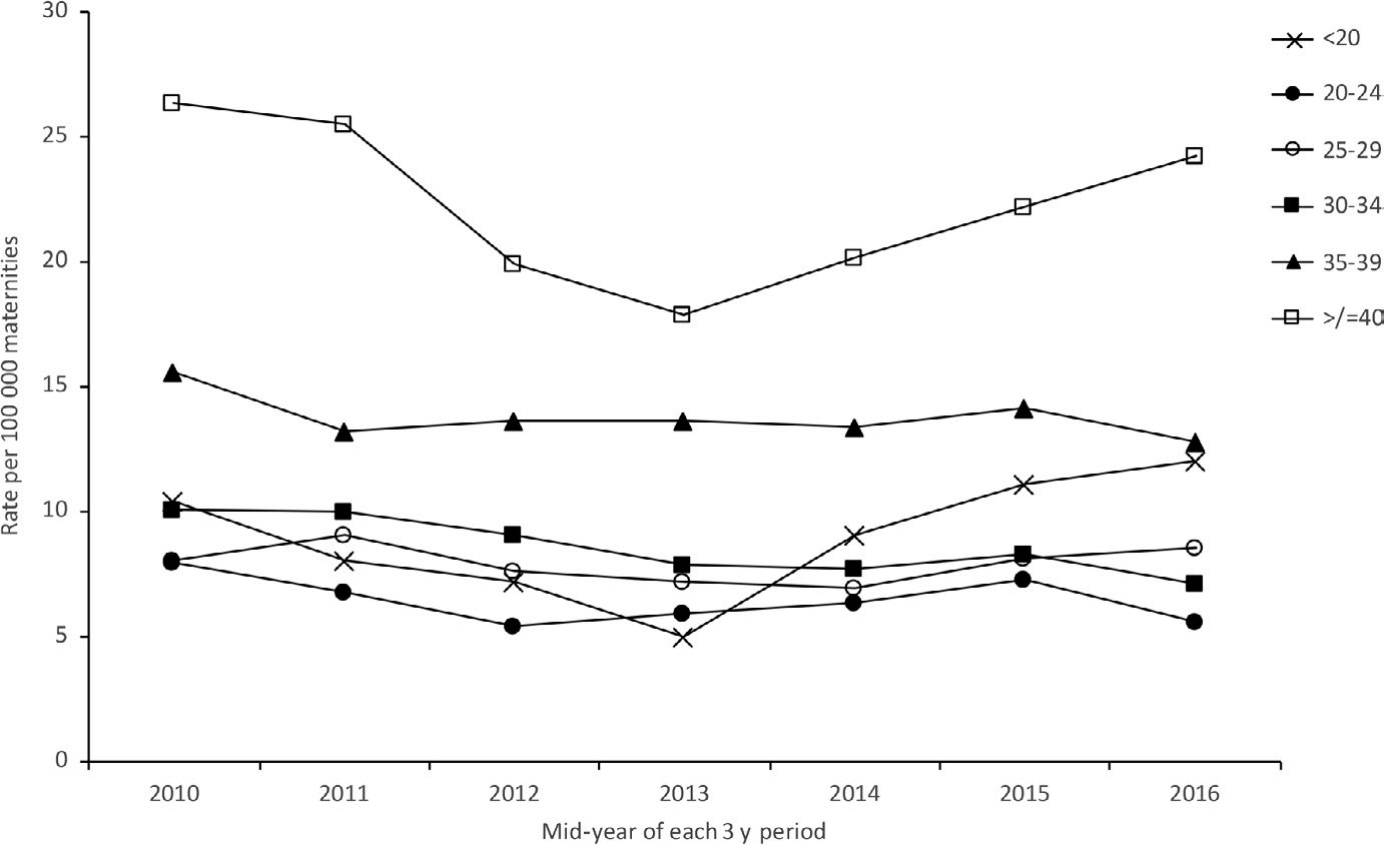
Maternal mortality amongst different age groups in the UK 2009‐17

**Figure 4:**
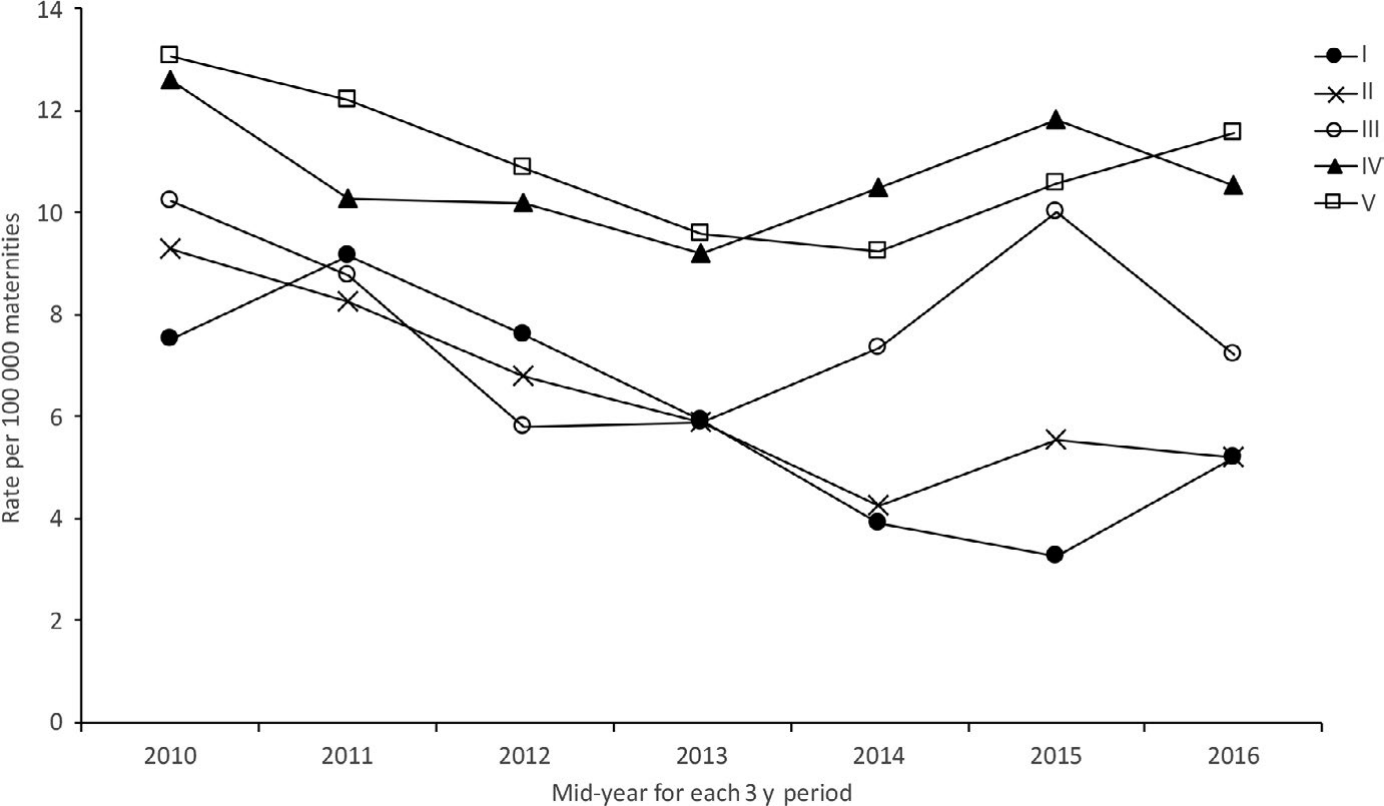
Maternal mortality rates by area deprivation score quintile for women's usual area of residence in England 2009‐17

In every 3‐year period, the patterns of disparity between the different population groups were similar (Figures 2, 3, 4, Table 1, Table S1, Figures S1‐S3). Women from black and Asian groups had a higher mortality rate than white women in most time periods, as did women aged 35 and over and women from the most deprived quintile areas of residence. There was evidence of an increasing trend in maternal mortality amongst black women (*P* = .029) and a decrease in mortality amongst women from IMD quintiles I (*P* = .054) and II (*P* = .029), but no trends over time in any of the other ethnic, age or IMD groups. There was a widening of the disparity between black and white women (RR 2.59 in 2009‐11 compared with 5.27 in 2015‐17, ratio of the relative risks [RRR] 2.03, 95% CI 1.11, 3.72; Table 1). There was no other evidence of widening disparities between population groups (Table 1).

**Table 1:** Relative risks of maternal mortality between different groups, and ratios of relative risks over time 2009‐17

	Relative risk (95% confidence interval)			Ratio of the relative risks (95% confidence interval)	
	2009‐11	2012‐14	2015‐17	2012‐14 with 2009‐11	2015‐17 with 2009‐11
Maternal age (y)					
<20	1.3 (0.65, 2.47)	0.84 (0.25, 2.25)	2.16 (0.86, 5.00)	0.65 (0.18, 2.34)	0.60 (0.20, 1.82)
20‐24	1.00 (Reference)	1.00 (Reference)	1.00 (Reference)	–	–
25‐29	1.01 (0.65, 1.59)	1.2 (0.72, 2.05)	1.53 (0.90, 2.74)	1.19 (0.60, 2.36)	1.51 (0.97, 2.37)
30‐34	1.26 (0.83, 1.95)	1.32 (0.81, 2.23)	1.27 (0.74, 2.27)	1.05 (0.54, 2.03)	1.01 (0.50, 2.04)
35‐39	1.95 (1.26, 3.03)	2.29 (1.38, 3.89)	2.30 (1.33, 4.11)	1.17 (0.60, 2.32)	1.18 (0.58, 2.41)
≥40	3.16 (1.79, 5.48)	3.00 (1.51, 5.83)	4.34 (2.26, 8.43)	0.95 (0.39, 2.28)	1.37 (0.58, 3.26)
IMD quintiles (England only)					
I (least deprived/highest 20%)	1.00 (Reference)	1.00 (Reference)	1.00 (Reference)	–	–
II	1.24 (0.69, 2.26)	0.99 (0.48, 2.05)	1.00 (0.46, 2.22)	0.80 (0.31, 2.04)	0.81 (0.31, 2.16)
III	1.25 (0.71, 2.24)	1.00 (0.50, 2.01)	1.39 (0.70, 2.90)	0.80 (0.32, 1.97)	1.11 (0.45, 2.77)
IV	1.65 (0.99, 2.84)	1.56 (0.86, 2.93)	2.03 (1.09, 4.01	0.95 (0.42, 2.12)	1.23 (0.53, 2.84)
V (most deprived/lowest 20%)	1.69 (1.03, 2.87)	1.62 (0.92, 2.99)	2.23 (1.23, 4.33	0.96 (0.44, 2.09)	1.32 (0.58, 2.97)
Ethnic group (England only)^a^					
White (inc. not known)	1.00 (Reference)	1.00 (Reference)	1.00 (Reference)	–	–
Asian	2.06 (1.45, 2.92)	1.36 (0.81, 2.18)	1.77 (1.10, 2.74)	0.66 (0.36, 1.21)	0.85 (0.48, 1.53)
Black	2.59 (1.67, 4.02)	4.19 (2.69, 6.35)	5.27 (3.44, 7.87)	1.62 (0.88, 2.99)	2.03 (1.11, 3.72)

Abbreviation: IMD, Index of Multiple Deprivation.

a Denominator data for women of mixed or Chinese/other ethnicity were not available in the same format for 2009‐11, therefore, 2012‐14 and 2015‐17 data could not be compared with 2009‐11.

Denominator data for women of mixed or Chinese/other ethnicity were not available in the same format for 2009‐11, therefore, 2012‐14 and 2015‐17 data could not be compared with 2009‐11. However, there was some evidence of an increasing disparity in mortality rate comparing mixed ethnicity women with white women between the two later triennia (RRR 7.09, 95% CI 0.39, 128.28 when comparing 2015‐17 with 2012‐14). Note there was no clear evidence of an increasing trend in mortality rate amongst women of mixed ethnicity over the period 2012‐17 (*P* = .083).

### 3.1 Comment

#### 3.1.1 Principal findings

There has been no overall change in the UK maternal mortality rate over the period 2009‐17. However, this analysis suggests there have been changes in specific population subgroups, with evidence that the maternal mortality rate is increasing amongst black women and decreasing amongst women resident in less deprived areas. There has been a widening of the disparity in mortality rate between black and white women, with black women experiencing a fivefold higher maternal mortality rate in 2015‐17, compared to a 2.5‐fold difference in 2009‐11 when compared to white women. There may also be a widening of the disparity in maternal mortality rate between women of mixed ethnicity and white women, with a threefold higher mortality rate in 2015‐17 compared to no difference in 2012‐14.

#### 3.1.2 Strengths of the study

This study used national data collected over 9 years with enhanced case ascertainment to identify maternal deaths, thus estimates of mortality rates in the whole population and within population subgroups are likely to be robust. Data on women's characteristics were collected from medical records or from published national vital statistics data and therefore have a high level of completeness and validity.

#### 3.1.3 Limitations of the data

Although this analysis uses national data collected over several years, maternal death remains an uncommon event. It is possible that some clinically important differences were not identified due to limited study power. The national confidential enquiry reports estimate denominator ethnicities by inclusion of any women with missing ethnic group information into the white ethnic group, since this most closely approximates the ethnic group distribution seen amongst birth registrations. It is possible therefore that there has been some misclassification of women's ethnicities in the denominator. This misclassification will not occur, however, amongst women who have died, since the information on ethnic group has been extracted from self‐reported ethnic group in women's medical records. Given changes to the ethnic groupings in which denominator data are published, the study was only able to examine trends over a period of 2012‐17 amongst women from mixed, Chinese or other ethnic groups, which further limits the power of the study to identify important trends. Denominator maternities data were not available at an individual level, therefore, an adjusted analysis was not possible.

#### 3.1.4 Interpretation

These findings suggest that there needs to be a particular targeting of research and action to address maternal mortality amongst black women in the UK, which appears to be increasing, and potentially also to address mortality amongst women of mixed ethnicity. In contrast, maternal mortality appears to be decreasing amongst women resident in less deprived areas. In England, a halving of maternal mortality between 2010 and 2030, subsequently revised to 2025, has been a national ambition since 2015.^24^ This analysis suggests that actions and policies put in place to date have been more successful in some population groups than others. The World Health Organisation has emphasised the importance of Health Equity Impact Assessment of all public policies,^25^, ^26^ and perhaps this consideration needs to be further emphasised in Maternal Death Surveillance and Response Programmes. A specific target focussing on ensuring continuity of midwifery care for black and minority ethnic women is one step in this direction.^27^

Previous studies in the UK have shown that pre‐existing medical co‐morbidities, anaemia during pregnancy, previous pregnancy problems, inadequate use of antenatal care, substance misuse, unemployment and maternal age are all independently associated with maternal death.^28^ Observed disparities between ethnic groups were attenuated after adjustment for gestational diabetes during the current pregnancy, medical co‐morbidities, previous pregnancy problems and inadequate use of antenatal care, thus it is possible that these factors explain some of the differences we observed amongst women from minority ethnic groups. However, there are a number of potential underlying factors which evaluation of quantitative data alone cannot elucidate, and a confidential enquiry approach,^29^ together with consideration of both implicit and explicit biases amongst both health systems and health professionals will be required.

A qualitative study of the maternity experiences of women with multiple disadvantages giving birth in England, most of whom were from black and minority ethnic groups, highlighted that women brought feelings of powerlessness and low self‐esteem to their maternity appointments, which could be made substantially worse by disrespectful care.^30^ Several women, particularly those from migrant backgrounds expressed confusion about the system of maternity care and their entitlements, which may explain differential take‐up of antenatal care, reflecting an effective lack of access even if health care is provided freely at the point of access. Other women emphasised the importance of a trusting relationship with their health care professional; respectful maternity care is recognised as a high priority to ensure safe motherhood^31^ and ensuring that this is provided appropriately to all population groups will be important to reduce inequity.

Similar disparities in maternal mortality have been identified in the USA, and addressing structural racism has been emphasised as one of the actions to begin to tackle this.^32^ Less attention has been given to this issue in the UK, although surveys of women's experiences of maternity care have reported that women from all minority ethnic groups report a poorer experience of care,^33^ and many women describe staff attitudes in largely negative terms.^34^ These studies do identify specific instances of structural factors, such as lack of availability of female doctors, which compromised access to care for women from minority ethnic groups.^34^ Other studies have identified adjustments needed in maternity services for women from other disadvantaged groups, such as those with learning disabilities^35^; these studies all identified that women who received specialised midwife care felt particularly able to disclose their concerns, allowing for potentially concerning medical complications to be recognised and treated.

## 4 CONCLUSIONS

The maternal mortality rate is static in the UK. This analysis has identified clear differences in the patterns of maternal mortality amongst different ethnic, age and socio‐economic groups in the UK. There appears to be no change in the patterns of disparity between most groups over time. However, there is evidence that the maternal mortality rate amongst black women in England is increasing and that the gap between black and white women in terms of their mortality rate is increasing. This emphasises the importance of research and policies focussed specifically on women from black and minority ethnic groups, together with other disadvantaged groups, to begin to reduce maternal mortality in the UK.

## CONFLICT OF INTEREST

All authors declare they have no conflicts of interest.
