# Supplementary material for: A national population‐based cohort study to investigate inequalities in maternal mortality in the United Kingdom, 2009‐17
Source: Paediatr Perinat Epidemiol. 2020 Feb 3;34(4):392–8. doi: 10.1111/ppe.12640 (PMC7383891; doi:10.1111/ppe.12640)
Supplement: Supplementary file 1 [file PPE-34-392-s001.docx]

| eTable 1: Maternal mortality in different population groups 2009-17 | | | | | | | | | | | | | | | | |
| --- | --- | --- | --- | --- | --- | --- | --- | --- | --- | --- | --- | --- | --- | --- | --- | --- |
|  | Age (years) | | | | | | IMD Quintiles (England only)* | | | | | Ethnic group (England only) | | | | |
|  | <20 | 20 – 24 | 25 – 29 | 30 – 34 | 35 – 39 | ≥ 40 | First quintile | Second quintile | Third quintile | Fourth quintile | Fifth quintile | White | Asian | Black | Chinese/ others | Mixed |
| 2009-11 |  |  |  |  |  |  |  |  |  |  |  |  |  |  |  |  |
| Total maternities | 135131 | 450958 | 657989 | 663761 | 379840 | 91250 | 292488 | 312027 | 361787 | 443189 | 543187 | 1585941 | 205103 | 93861 | NA | NA |
| Total deaths | 14 | 36 | 53 | 67 | 59 | 24 | 22 | 29 | 37 | 56 | 71 | 150 | 40 | 23 | NA | NA |
| Rate per 100,000 maternities (95% CI) | 10.36 (5.66, 17.38) | 7.98 (5.59, 11.05) | 8.05 (6.03, 10.54) | 10.09 (7.82, 12.82) | 15.53 (11.82, 20.04) | 26.30 (16.85, 39.13) | 7.52 (4.71, 11.39) | 9.29 (6.22, 13.35) | 10.23 (7.20, 14.10) | 12.64 (9.55, 16.41) | 13.07 (10.21, 16.49) | 9.46 (8.01, 11.10) | 19.50 (13.93, 26.56) | 24.50 (15.53, 36.77) | NA | NA |
| Relative risk (95% CI) | 1.30 (0.70, 2.41) | 1.00 (reference) | 1.01 (0.66, 1.54) | 1.26 (0.84, 1.90) | 1.95 (1.29, 2.95) | 3.29 (1.97, 5.52) | 1.00 (reference) | 1.24 (0.71, 2.15) | 1.36 (0.80, 2.30) | 1.68 (1.03, 2.75) | 1.74 (1.08, 2.80) | 1.00 (reference) | 2.06 (1.45, 2.92) | 2.59 (1.67, 4.02) | NA | NA |
| 2010-12 |  |  |  |  |  |  |  |  |  |  |  |  |  |  |  |  |
| Total maternities | 124576 | 446261 | 666023 | 690459 | 379992 | 94205 | 295264 | 314987 | 365220 | 447395 | 548341 | 1599567 | 210452 | 91593 | NA | NA |
| Total deaths | 10 | 30 | 60 | 69 | 50 | 24 | 27 | 26 | 32 | 46 | 67 | 144 | 39 | 25 | NA | NA |
| Rate per 100,000 maternities (95% CI) | 8.03 (3.85, 14.76) | 6.72 (4.54, 9.60) | 9.01 (6.87, 11.60) | 9.99 (7.78, 12.65) | 13.16 (9.77, 17.35) | 25.48 (16.32, 37.90) | 9.14 (6.03, 13.30) | 8.25 (5.39, 12.09) | 8.76 (5.99, 12.37) | 10.28 (7.53, 13.71) | 12.22 (9.47, 15.51) | 9.00 (7.59, 10.60) | 18.53 (13.18, 25.33) | 27.29 (17.66, 40.29) | NA | NA |
| Relative risk (95% CI) | 1.19 (0.58, 2.44) | 1.00 (reference) | 1.34 (0.86, 2.08) | 1.49 (0.97, 2.28) | 1.96 (1.24, 3.08) | 3.79 (2.22, 6.48) | 1.00 (reference) | 0.90 (0.53, 1.55) | 0.96 (0.57, 1.60) | 1.12 (0.70, 1.81) | 1.34 (0.85 2.09) | 1.00 (reference) | 2.06 (1.45, 2.93) | 3.03 (1.98, 4.64) | NA | NA |
| 2011-13 |  |  |  |  |  |  |  |  |  |  |  |  |  |  |  |  |
| Total maternities | 111805 | 427329 | 662206 | 701163 | 374999 | 95607 | 291274 | 310685 | 361339 | 442527 | 543159 | 1578959 | 211934 | 86737 | NA | NA |
| Total deaths | 8 | 23 | 50 | 63 | 51 | 19 | 22 | 21 | 21 | 45 | 59 | 126 | 39 | 23 | NA | NA |
| Rate per 100,000 maternities (95% CI) | 7.16 (3.09, 14.10) | 5.38 (3.41, 8.08) | 7.55 (5.60, 9.95) | 8.99 (6.90, 11.50) | 13.60 (10.13, 17.88) | 19.87 (11.97, 31.03) | 7.55 (4.73, 11.44) | 6.76 (4.18, 10.33) | 5.81 (3.60, 8.88) | 10.17 (7.42, 13.61) | 10.86 (8.27, 14.01) | 7.98 (6.65, 9.50) | 18.40 (13.09, 25.16) | 26.52 (16.81, 39.79) | NA | NA |
| Relative risk (95% CI) | 1.33 (0.51, 3.08) | 1.00 (reference) | 1.40  (0. 84, 2.41) | 1.67 (1.02, 2.82) | 2.53 (1.52, 4.33) | 3.69 (1.90, 7.09) | 1.00 (reference) | 0.89 (0.47, 1.71) | 0.77 (0.40, 1.47) | 1.35 (0.79, 2.35) | 1.44 (0.87, 2.46) | 1.00 (reference) | 2.31 (1.61, 3.30) | 3.32 (2.13, 5.18) | NA | NA |

|  | AGE (YEARS) | | | | | | IMD QUINTILES (ENGLAND ONLY)* | | | | | ETHNIC GROUP (ENGLAND ONLY) | | | | |
| --- | --- | --- | --- | --- | --- | --- | --- | --- | --- | --- | --- | --- | --- | --- | --- | --- |
|  | <20 | 20 – 24 | 25 – 29 | 30 – 34 | 35 – 39 | ≥ 40 | First quintile | Second quintile | Third quintile | Fourth quintile | Fifth quintile | White | Asian | Black | Chinese/ others | Mixed |
| 2012-14 |  |  |  |  |  |  |  |  |  |  |  |  |  |  |  |  |
| Total maternities | 100014 | 403278 | 657886 | 710835 | 374528 | 95083 | 286878 | 305930 | 355782 | 433834 | 531779 | 1550562 | 207975 | 93151 | 72976 | 30138 |
| Total deaths | 5 | 24 | 47 | 56 | 51 | 17 | 17 | 18 | 21 | 40 | 51 | 115 | 21 | 29 | 3 | 1 |
| Rate per 100,000 maternities (95% CI) | 5.00 (1.62, 11.67) | 5.95 (3.81, 8.85) | 7.14 (5.25, 9.50) | 7.88 (5.95, 10.23) | 13.62 (10.14, 17.90) | 17.88 (10.42, 28.60) | 5.93 (3.45, 9.49) | 5.88 (3.49 to 9.30) | 5.90 (3.65, 9.02) | 9.22 (6.59, 12.55) | 9.59 (7.14, 12.61) | 7.42 (6.12, 8.90) | 10.10 (6.25, 15.43) | 31.13 (20.80, 44.70) | 4.11 (0.84, 12.01) | 3.32 (0.08, 18.49) |
| Relative risk (95% CI) | 0.84 (0.25, 2.25) | 1.00 (reference) | 1.20 (0.72, 2.05) | 1.32 (0.81, 2.23) | 2.29 (1.38, 3.89) | 3.00 (1.51, 5.83) | 1.00 (reference) | 0.99 (0.48, 2.05) | 1.00 (0.50, 2.01) | 1.56 (0.86, 2.93) | 1.62 (0.92, 2.99) | 1.00 (reference) | 1.36 (0.81, 2.18) | 4.19 (2.69, 6.35) | 0.55 (0.11, 1.66) | 0.44 (0.01, 2.54) |
| 2013-15 |  |  |  |  |  |  |  |  |  |  |  |  |  |  |  |  |
| Total maternities | 88846 | 376968 | 652426 | 712524 | 380872 | 94198 | 280943 | 305341 | 352761 | 428041 | 520239 | 1535033 | 203888 | 88743 | 73736 | 30254 |
| Total deaths | 8 | 24 | 45 | 55 | 51 | 19 | 11 | 13 | 26 | 45 | 48 | 101 | 22 | 25 | 3 | 3 |
| Rate per 100,000 maternities (95% CI) | 9.00 (3.89, 17.74) | 6.37 (4.08, 9.47) | 6.90 (5.03, 9.23) | 7.72 (5.82, 10.05) | 13.39 (9.97, 17.61) | 20.17 (12.14, 31.50) | 3.92 (1.95, 7.01) | 4.26 (2.27, 7.28) | 7.37 (4.81, 10.80) | 10.51 (7.67, 14.07) | 9.23 (6.80, 12.23) | 6.58 (5.36, 7.99) | 10.79 (6.76, 16.34) | 28.17 (18.23, 41.60) | 4.07 (0.83, 11.89) | 9.92 (2.04, 28.98) |
| RATE 95% CI |  |  |  |  |  |  |  |  |  |  |  |  |  |  |  |  |
| Relative risk (95% CI) | 1.41 (0.55, 3.25) | 1.00 (reference) | 1.08 (0.65, 1.86) | 1.21 (0.74, 2.05) | 2.10 (1.27, 3.57) | 3.17 (1.64, 6.03) | 1.00 (reference) | 1.09 (0.45, 2.68) | 1.88 (0.90, 4.22) | 2.69 (1.37, 5.76) | 2.36 (1.21, 5.03) | 1.00 (reference) | 1.64 (0.98, 2.62) | 4.28 (2.65, 6.69) | 0.62 (0.13, 1.86) | 1.51 (0.31, 4.53) |
| 2014-16 |  |  |  |  |  |  |  |  |  |  |  |  |  |  |  |  |
| Total maternities | 81185 | 357941 | 651355 | 720962 | 395374 | 94723 | 276162 | 306896 | 349005 | 422310 | 510542 | 1529881 | 199661 | 85735 | 75235 | 30639 |
| Total deaths | 9 | 26 | 53 | 60 | 56 | 21 | 9 | 17 | 35 | 50 | 54 | 123 | 29 | 34 | 4 | 5 |
| Rate per 100,000 maternities (95% CI) | 11.10 (5.07, 21.04) | 7.26 (4.74, 10.64) | 8.14 (6.10, 10.64) | 8.32 (6.35, 10.71) | 14.16 (10.70, 18.39) | 22.17 (13.72, 33.89) | 3.26 (1.49, 6.19) | 5.54 (3.23, 8.87) | 10.03 (6.99, 13.95) | 11.84 (8.79, 15.61) | 10.58 (7.95, 13.80) | 8.04 (6.68, 9.59) | 14.52 (9.73, 20.86) | 39.66 (27.47, 55.41) | 5.32 (1.45, 13.61) | 16.32 |
| Relative risk (95% CI) | 1.53 (0.63, 3.36) | 1.00 (reference) | 1.12 (0.69, 1.87) | 1.15 (0.71, 1.89) | 1.95 (1.20, 3.24) | 3.05 (1.63, 5.64) | 1.00 (reference) | 1.70 (0.72, 4.33) | 3.08 (1.45, 7.28) | 3.63 (1.77, 8.40) | 3.25 (1.59, 7.48) | 1.00 (reference) | 1.81 (1.16, 2.73) | 4.93 (3.27, 7.26) | 0.66 (0.18, 1.74) | 2.03 (0.65, 4.87) |

|  | Age (years) | | | | | | IMD Quintiles (England only)* | | | | | Ethnic group (England only) | | | | |
| --- | --- | --- | --- | --- | --- | --- | --- | --- | --- | --- | --- | --- | --- | --- | --- | --- |
|  | <20 | 20 – 24 | 25 – 29 | 30 – 34 | 35 – 39 | ≥ 40 | First quintile | Second quintile | Third quintile | Fourth quintile | Fifth quintile | White | Asian | Black | Chinese/ others | Mixed |
| 2015-17 |  |  |  |  |  |  |  |  |  |  |  |  |  |  |  |  |
| Total maternities | 74837 | 340586 | 642794 | 721328 | 405959 | 94920 | 269945 | 308115 | 346150 | 418292 | 500949 | 1523822 | 196199 | 84144 | 75442 | 31104 |
| Total deaths | 9 | 19 | 55 | 51 | 52 | 23 | 14 | 16 | 25 | 44 | 58 | 110 | 25 | 32 | 7 | 7 |
| Rate per 100,000 maternities (95% CI) | 12.03 (5.50, 22.83) | 5.58 (3.36, 8.71) | 8.56 (6.45, 11.14) | 7.07 (5.26, 9.30) | 12.81 (9.57, 16.80) | 24.23 (15.36, 36.36) | 5.19 (2.84, 8.70) | 5.19 (2.97, 8.43) | 7.22 (4.67, 10.66) | 10.52 (7.64, 14.12) | 11.58 (8.79, 14.97 | 7.22 (5.93, 8.70 | 12.74 (8.25, 18.81) | 38.03 (26.01, 53.68) | 9.28 (3.73, 19.12) | 22.51 (9.05, 46.36) |
| Relative risk (95% CI) | 2.16 (0.86, 5.00) | 1.00 (reference) | 1.53 (0.90, 2.74) | 1.27 (0.74, 2.27) | 2.30 (1.33, 4.11) | 4.34 (2.26, 8.43) | 1.00 (reference) | 1.00 (0.46, 2.22) | 1.39 (0.70, 2.90) | 2.03 (1.09, 4.01) | 2.23 (1.23, 4.33) | 1.00 (reference) | 1.77 (1.10, 2.74) | 5.27 (3.44, 7.87) | 1.29 (0.50, 2.74) | 3.12 |

*First quintile = least deprived

CI = Confidence interval

IMD = Index of Multiple Deprivation

eFigure S1: Maternal mortality rates with 95% confidence intervals amongst different ethnic groups in England 2009-17

Note denominator data for mixed and Chinese/other ethnic groups are not available for 2009-11 therefore data are only presented from 2012-14 onwards

eFigure S2: Maternal mortality rates with 95% confidence intervals amongst different age groups in the UK 2009-17

eFigure S3: Maternal mortality rates with 95% confidence intervals by area deprivation score quintile for women’s usual area of residence in England 2009-17
